# A comment on: A non-randomized clinical trial to determine the safety and efficacy of a novel sperm sex selection technique

**DOI:** 10.1371/journal.pone.0282208

**Published:** 2023-03-01

**Authors:** Guido Pennings

**Affiliations:** Department of Philosophy and Moral Science, Bioethics Institute Ghent (BIG), Ghent University, Gent, Belgium; ANZAC Research Institute, AUSTRALIA

To the Editor,

Sex selection is still a highly controversial technique. This Comment is not the place to present the arguments for or against this application. Instead it focuses on the reasons why the authors believe their method to be more ethically palatable. The landscape of sex selection has changed considerably in recent years due to technical developments. In the United States, more than 40% of all IVF cycles involved PGT-A [1]. The Ethics Committee of the American Society for Reproductive Medicine advises clinics to inform patients that the sex of resulting embryos may be known as part of embryo testing and to ask them whether they wish to receive this information [2]. So infertility patients may find out the sex of their embryos and request the transfer of an embryo of the sex they desire. There is a difference between those who start an IVF cycle solely for sex selection and those who perform an IVF cycle for infertility reasons and request transfer of an embryo of the desired sex when a euploid embryo is available. For the latter, sex selection after PGT-A is an extra advantage, not a goal. A large majority of American fertility clinics offers non-medical sex selection to their patients [3].

The authors several times refer to the ethical nature of their new technique. They state that embryo selection techniques are controversial, presumably because of the moral status of the embryo. To avoid this problem, they propose their sperm selection technique as more ethically acceptable. They consider their method as a stand-alone method, independent from IVF/PGT-A. This implies that the technique has to be combined with intrauterine insemination (IUI). In this article, no information is included about this change of methods. Future research will have to demonstrate that the enriched sperm samples can be used in combination with IUI and that a similar efficacy can be obtained.

The method used for the selection of the sex is important when the indication for selection is taken into account. The sperm selection technique in combination with IUI would be morally acceptable for elective sex selection even when there is a 20% risk of obtaining the wrong sex. However, this would not be the case when there is a high genetic risk. In that case, IVF/PGT-A would be necessary. The sperm selection technique would be useful in combination with IVF since this would result in a higher proportion of embryos of the desired sex. This skewed sex ratio would increase the possibility of finding a good quality embryo of the sex without an high genetic risk. Still, it remains to be seen whether twenty percent risk of the wrong sex in elective sex selection situations is acceptable to many people. For those who have the financial capacity to pay for an IVF cycle with PGT-A, this risk is likely too high. Moreover, the argument that embryo selection is ethically contentious may be correct in the larger population but it is unlikely to be decisive for both fertility patients and fertility doctors. Everyone involved in applying standard IVF protocols needs to accept the creation and destruction of (healthy) embryos.

The authors rightly point out that safety and efficacy of a method are crucial. The authors should be applauded for including a follow-up study on the health of the offspring. No minor or major malformations were found and no developmental delays at the age of 3. Follow-up studies in the field of medically assisted reproduction are rare [4]. Although new techniques are constantly introduced into this field, very few fertility centres make the effort to check on the effect in terms of efficacy and health. The number of offspring born after the sperm selection technique is too small (n = 29) to draw a reliable conclusion at this stage but it is a good start. Continuation of this follow-up after the move to IUI is needed to obtain more certainty. Moreover, the authors also compared the aneuploidy rate of the embryos of the study and the control group and found no statistically significant difference. The second element is the efficacy. With a 80% enriched sperm sample for both sexes, this method scores among the most efficacious methods of sperm selection. The additional control of the sex of the resulting embryos confirms the efficacy of the method. Such information is needed not only to justify the application of this new technology but also to be able to properly inform the patients. Still, if they indeed intend this method to be used without PGT, confirmation of efficacy in IUI is needed.

The authors also claim that this technique will minimize embryo wastage. This remark only makes sense within the context of IVF/PGT-A. When no sperm sex selection is used, approximately 50% of the embryos will be of the desired sex. The number of embryos of the desired sex increases with the technique but that does not imply that fewer embryos will be discarded. When the first transfer is successful, the couple may not come back for a second child and the remaining embryos may be destroyed, including those of the desired sex. To determine whether or not embryo wastage has indeed be avoided, data are needed on what happens in the long run with the cryopreserved embryos after the birth of a first child with the desired sex. In fact, there would only be fewer embryos wasted if the high efficacy of the method would allow fewer embryos to be created.

Studies indicate that the majority of the people choosing to have an IVF cycle to select the sex of their child want to balance their family. Most of them already have 2 or 3 children of the same sex [5]. The reasons underlying family balancing refer to the wish to raise a child of the other sex, to increase of dynamics in the family and to raise a child of the same gender as the parent. These motives make it unlikely that these people will come back for a second child of the same sex. It is important to inform patients that the application of this technique may be counterproductive if they later desire a child of the opposite sex. Bayefsky and colleagues found that about 80% of patients who had embryos frozen of which the sex was known after PGT-A (but who had not started the cycle for sex selection as the main purpose) selected the opposite sex of the first life birth for the second child [6]. If couples who had a first birth of the desired sex would want a second child of the other sex, they would have to work with approximately 20% of the remaining embryos, thus risking transfer of low quality embryos.

The present study did not find a lower implantation rate or clinical pregnancy rate between the sex selection group and the control group. This may be explained by the efficacy of the method in creating more embryos of the desired sex. In many cases of PGT-A for sex selection, however, a choice has to be made between the best quality embryo and an embryo of the desired sex. Two recent studies both indicated a significantly reduced implantation rate when couples selected the embryo on the basis of desired sex rather than on best quality [7, 8]. The new method may decrease the number of possible conflicts between quality and desired sex because of the higher proportion of embryos of the desires sex. In the present study, a lower quality embryo of the desired sex was replaced instead of a better quality embryo of the other sex in a small number of cycles (n = 5) but all of them still resulted in a term pregnancy (personal communication). It seems to be within the logic of the request that those who started the IVF cycle in order to obtain a child with a specific sex will put priority on the sex rather than on the success rate of the cycle.

In summary, the present study investigates the safety and efficacy of a sperm sex selection method in a PGT-A setting. This study allows the collection of information that is essential to move to the utilization of the method in combination with IUI.

## References

[1] Eaton J.L. State-mandated in vitro fertilization coverage and utilization of preimplantation genetic testing. Obstet Gynecol 2022;139:498–499. 35271542 10.1097/AOG.0000000000004737

[2] Ethics Committee of the American Society for Reproductive Medicine. Disclosure of sex when incidentally revealed as part of preimplantation genetic testing (PGT): an Ethics Committee opinion. Fertil Steril 2018;110:625–627. doi: 10.1016/j.fertnstert.2018.06.019 30196948

[3] Capelouto SM, Archer SR, Morris JR, Kawwass JF, Hipp HS. Sex selection for non-medical indications: a survey of current pre-implantation genetic screening practices among U.S. ART clinics. J Assist Reprod Gen 2018;35:409–416. doi: 10.1007/s10815-017-1076-2 29080968 PMC5904054

[4] Jans V, Dondorp W, Bonduelle M, de Die C, Mertes H, et al. Follow-up in the field of reproductive medicine: an ethical exploration. Reprod Biomed Online 2020;41:1144–1150. doi: 10.1016/j.rbmo.2020.08.033 32967810

[5] Bracewell-Milnes T, Saso S, Jones B, Cato S, Parikh R, et al. A systematic review exploring the patient decision-making factors and attitudes towards pre-implantation genetic testing for aneuploidy and gender selection. Acta Obstet Gynecol Scand 2021;100:17–29. doi: 10.1111/aogs.13973 32862440

[6] Bayefsky M, Martel RA, Hamer D, Shaw J, Blakemore JK. A balancing act: sex selection after pre-implantation genetic testing for aneuploidy (PGT-A) for first versus second baby. Fertil Steril 2021;16(3)(suppl):e70–e71. 10.1093/humrep/dead101 37208860

[7] Arnold A, Henry L, Lee R, McReynolds S, Schoolcraft WB, et al. Non-medical embryo selection results in reduced implantation compared to the transfer of the highest morphological grade blastocysts. Fertil Steril 2022;118(4) (suppl): e269.

[8] Gill P, Whitehead CV, Reig A, Margolis CK, Roberts LM, et al. Best quality vs sex selection–what takes priority for patients undergoing PGT-A? A historical analysis of embryo selection preferences over a ten-year period. Fertil Steril 2022;118(4)(suppl):e268–e269.

